# Cholera

**Published:** 1850-03

**Authors:** 


### CHOLERA.

We regret to learn that cholera is prevailing to some extent in New Orleans. The official reports announce one hundred and eleven deaths, during the month of December, from that disease. We learn, also, that steamboats leaving New Orleans often have cases on board, especially among the emigrants. A few cases are reported to have occurred in St. Louis. Cincinnati, thus far, remains free from the disease.—*Western Lancet.*

